# Assessing the health-related quality of life in informal caregivers of Alzheimer’s Disease: evidence from Malaysia

**DOI:** 10.1186/s12904-025-01943-8

**Published:** 2025-12-01

**Authors:** Lyn Xuan Tay, Siew Chin Ong, Hui Ming Ong, Ewe Eow Teh, Alan Swee Hock Ch’ng, Ing Khieng Tiong, Rizah Mazzuin Razali, Thaigarajan Parumasivam

**Affiliations:** 1 Discipline of Social and Administrative Pharmacy, Universiti Sains Malaysia, Georgetown, Pulau Pinang 11800 Malaysia; 2 Department of Psychiatry and Mental Health, Hospital Pulau Pinang, Ministry of Health Malaysia, Georgetown, Pulau Pinang 10990 Malaysia; 3 Department of Medicine, Seberang Jaya Hospital, Ministry of Health Malaysia, Seberang Perai, Penang 13700 Malaysia; 4 Department of Geriatric, Pusat Jantung Sarawak, Ministry of Health Malaysia, Kota Samarahan, Sarawak 94300 Malaysia; 5 Geriatric Unit, Department of Medicine, Kuala Lumpur Hospital, Ministry of Health Malaysia, Kuala Lumpur, 50586 Malaysia; 6 Discipline of Pharmaceutical Technology, Universiti Sains Malaysia, Georgetown, Pulau Pinang 11800 Malaysia

**Keywords:** Alzheimer, Dementia, Well-being, Quality of life, Informal caregiver, SF-36

## Abstract

**Background:**

There were limited local studies to examine the health-related quality of life (HRQoL) in informal caregivers of patients with Alzheimer’s disease (AD) in Malaysia. This study aimed to evaluate the HRQoL in informal caregivers of AD along with its predictors.

**Materials and methods:**

132 complete responses were obtained from informal caregivers of patients with AD recruited in 4 tertiary hospitals during outpatient visits. Their HRQoL was assessed with the 36-item Short Form Health Survey (SF-36). Sociodemographic of both patients and caregivers were assessed as well as the time spent in informal care via a structured questionnaire. Summary scores of each SF-36 domains, Physical Component Summary (PCS) and Mental Component Summary (MCS) were compared with population norm in Malaysia. Forward stepwise multiple linear regression was conducted to identify significant factors influencing caregivers’ HRQoL.

**Results:**

With the mean PCS (51.19 ± 9.25) and MCS (44.17 ± 11.19), a declining trend was observed along with increasing disease severity in the study population. Besides the domain of physical functioning, a significant decline was detected in all remaining seven SF-36 domains compared to Malaysia general population. In multivariable models, demand in basic activity of daily living (BADL) (standardised β=-0.25, *p* = 0.002) and increasing caregivers’ age (standardised β=-0.30, *p* < 0.001) were negatively correlated with PCS. Female caregivers tend to attain lower PCS (standardised β=-0.19, *p* = 0.017). Nevertheless, providing care to female patients was related with higher MCS (standardised β = 0.23, *p* = 0.008). .

**Conclusion:**

Informal caregivers of patients with AD demonstrated diminished overall well-being regardless of physical or mental aspects. These findings underscore the necessity for support programs that address physical care demands and provide psychosocial interventions, particularly for older and female caregivers.

## Introduction

As one of the major etiologies in dementia, Alzheimer’s disease (AD) is characterised by progressive cognitive and functional decline [1–3]. It has affected 55 million individuals in 2022 and expected to become triple (152.8 million) in next 30 years along with the demographic shift towards aging population [4, 5]. From an economic perspective, $16.9 trillion expenditure is forecasted on the resource use in treatment and social care for Alzheimer’s disease and related dementia (ADRD) where low-middle income countries (LMIC) occupied two-third of them [6]. In the UK, the cost of dementia was estimated to double from 11.7 billion GBP to 23.5 billion GBP in 2050 along with 18% increase in population aged 65 above [7]. In Malaysia, the prevalence of dementia was found to be 8.5% from a national representative sample where the annual economic burden of AD was recently reported to be USD 8619 per capita from a societal perspective [8, 9]. As it increases with disease severity, indirect cost, quantified by informal care, still remains as the largest cost proportion (50–70%) in estimating the economic burden of AD [10]. Considering its impact towards aging society, AD is recognised as a worldwide public health concern clinically, economically and socially [7, 11]. 

Knowing that there is no cure for AD, informal care is one core component in AD which is mainly borne by family carers. In most studies, family carers such as spouse, adult children and siblings takes up the responsibility to provide daily care for AD patients in activities of daily living (ADL) such as bathing, eating, managing finance, doing chores and supervision [12, 13]. Such practice is observed as a family tradition representing filial piety particularly in Asian societies [14–17]. However, long caregiving hours in AD leads to negative consequences in caregivers health related quality of life (HRQoL) such as physical fatigue, psychological stress, emotional burnout, social isolation and deteriorating health conditions [14–16, 18–22]. Such scenario is worse among spousal caregivers due to their increasing age and multiple comorbidities [14, 23, 24]. With that, assessment to caregivers’ well-being is a rising concern in AD care considering the gradual increase in dementia prevalence along with population aging.

The concept of long-term care management is utmost important in relation with HRQoL. As there are different definitions across literatures, quality of life (QoL) is viewed in multiple dimensions such as psychological state, physical health, level of independence, personal beliefs and spirituality, social relationships and environment [25]. By incorporating the concept of health status, HRQoL refers to the components of QoL that could be influenced by health, disease, disorder and injury directly or indirectly [19, 26]. In AD patients, HRQoL significantly declines with multiple factors including disease progression, functional dependence and behavioural psychological symptoms of dementia (BPSD) [19, 27–31]. Recent local evidence also revealed the marked decline of HRQOL in patients with AD which was measured via proxy using both EuroQol 5-Dimensions (EQ-5D) and Quality-of-Life Alzheimer’s Disease (QOL-AD) instruments [32]. Being the main pillar of support for AD patients, informal caregivers also reported poor HRQoL compared to non-dementia caregivers with greater burden, stress, anxiety and depression [22, 27, 33–37]. Therefore, the subjective well-being of local caregivers should be investigated as an integral part in understanding the long-term AD caregiving landscape in Malaysia.

Investigating the impact of informal care towards caregivers physical and mental HRQoL could assist healthcare policymakers to make informed decision in ageing and geriatric care. With that, various generic HRQoL instruments such as EQ-5D and Medical Outcomes Study Questionnaire Short Form 36 health survey (SF-36) were applied in previous studies to evaluate caregivers’ perceptions in well-being [18, 22, 23, 27, 38–41]. Among these instruments, SF-36 evaluates HRQoL in eight dimensions and further composited into physical and mental summary scores [42–44]. With its ability to explore physical and mental aspects of HRQoL, it is useful in recognising potential effectiveness in new intervention in dementia care [35]. In Japan, diminished HRQoL was observed in their study population using the SF-36 instrument where similar findings were found in other countries [27, 33, 38, 40, 41, 45]. 

In a multinational study, AD caregivers from Japan scored greater in both physical and mental well-being compared to western countries such as USA, Spain and Italy [22]. This could be attributed to the lower depression, anxiety and care burden in Japan society which was indirectly influenced by national policies in long-term care institutions [22, 41]. On the other side, AD caregivers in some western countries experienced rising care burden with diminished well-being due to cultural values, limited access and high cost to respite-care support [22]. Unlike health policies that encourage community-based AD care in western countries, national policies on long-term care insurance was well-established in Japan to address challenges in population aging and healthcare sustainability [46]. In Malaysia, there are limited studies that investigates the HRQOL in informal caregiver of AD using standardised health utility measure. For instance, Nasreen et al. utilized the Control, Autonomy, Self-Realization, and Pleasure Scale (CASP-19) to measure the well-being of family caregivers of dementia [36]. Compared to health utility tools such as SF-36 and EQ-5D, it has limited capacity to reflect societal preferences for various health states [47]. A cross-sectional study was conducted among dementia caregivers in Sarawak (East Malaysia) using the SF-36 instrument. Despite the advantage of enabling cross-comparison with other country cohorts, the recruitment from a single state in Malaysia limited its representativeness of the national AD community.

In order to address the issue of limited evidence in carer HRQoL, it is encouraged to obtain empirical evidence across countries [48]. Such patient reported outcomes could help us understand the important factors affectinglocal carers' perception of well-being in providing care to AD patients [14, 18, 36]. As informal care still remains as the major component in AD care, HRQoL was widely applied in determining choice of treatments and efficacy of new interventions. By exploring the factors affecting the carers HRQoL in both physical and mental aspects, interventions and care management in AD could be designed with tapered needs among caregivers with different settings [15, 20, 35, 48]. Not only that, healthcare providers and stakeholders could formulate appropriate care plan and proper referral to accommodate the multidimensional demands from caregiving by identifying significant modifiable factors in local context [14, 19]. With that, the objective of this study is to evaluate the HRQoL among informal caregivers of AD in both physical and mental well-being along with their predictors.

## Methodology

### Study design

This study was a cross-sectional study conducted at four tertiary hospitals in Malaysia: Pusat Jantung Sarawak (PJS), Hospital Pulau Pinang (HPP), Hospital Seberang Jaya (HSJ), and Hospital Kuala Lumpur (HKL). As there are geriatricians or psychiatry specialists stationed in Northern (HPP, HSJ) Central (HKL) and East (PJS) Malaysia, convenience sampling was conducted in these 4 selected study sites. The data collection procedure was executed in three distinct phases: (1) participant recruitment and screening, (2) data abstraction from medical records, and (3) caregiver interview. To be eligible to this study, informal caregivers were the ones taking care of patients aged 65 years and above with a clinical diagnosis of AD according to The Diagnostic and Statistical Manual of Mental Disorders (DSM 5th Edition, DSM-V) [49], and attended outpatient visits in study sites during the study period. In addition, they were at least 18 years and above and responsible for the informal care for more than 3 months. Regarding those patients without regular follow up (missed more than 2 follow-up sessions) in the same hospital or those who participated in an interventional clinical trial in the post-index period, they were excluded in the study. Recruitment was done by specialists or the principal investigators, along with the patients’ respective informal caregivers from 1 January 2023 to 31 December 2023. The prevalence approach was applied to calculate the required sample size by using the Scalex SP calculator [50]. For the expected prevalence of 8.5% from the National Health Morbidity Survey 2018 Elderly Health Volume 2 [8], with an absolute precision of ± 5% and a potential loss/attrition of 10%, the required sample size was 134 [50, 51]. 

### Procedures

Followed by recruitment, written informed consent was granted by informal caregivers for data collection and interview. Subsequently, demographic and clinical data of patients with AD were collected retrospectively from patient medical records Next, an interview was held with respective informal caregivers to collect sociodemographic data of primary caregivers via structured questionnaire. Types of daily assistance and time spent on informal care were also collected via structures questionnaire adapted from the instrument Resource Use in Dementia (RUD) [52]. To assess informal caregivers’ HRQoL, Short Form-36 Health Survey (SF-36), developed by the Medical Outcome Study (MOS) was utilized in this interview. Ethical approval was obtained from the Medical Research Ethics Committee (MREC) Malaysia (NMRR-ID-21–02014-VCP (IIR)).

### Instruments

Information of AD patients were sought from patient medical records such as age, ethnicity, gender, disease severity (based on DSM-V), cognitive status (assessed by Mini-Mental State Examination (MMSE)) and presence of BPSD. In the structured questionnaire, demographics of primary caregivers such as age, ethnicity gender, relationship to patient, marital status, cohabitation status, number of informal caregivers involved, employment status, presence of medical comorbidities and income range were collected. The income level was stratified based on the National Salary and Wages Report 2022, which defines three income groups: B40 (Bottom 40%), M40 (Middle 40%), and T20 (Top 20%), with respective income thresholds in RM as outlined in the report [53]. Adapted from the RUD instrument, time spent on informal care for ADL was assessed in three aspects namely Basic ADL (BADL), Instrumental ADL (IADL) and supervision (SV) in monthly basis [13]. 

In this study, SF-36 instrument was employed to assess the HRQoL scores of primary caregivers. The SF-36 is a well-known and widely used health status measure that evaluates two crucial dimensions of quality of life: physical and mental health [54]. The eight multi-item scales include physical functioning (PF), physical role (RP), bodily pain (BP), general health (GH), vitality (VT), social functioning (SF), emotional role (RE), and mental health (MH). The physical component summary (PCS) comprises the first four scales under the physical health dimension, while the mental component summary (MCS) includes the last four scales under the mental health dimension [43, 55]. To facilitate comparisons across different populations, each scale’s scores were converted to a norm-based scoring system with a mean of 50 and a standard deviation of 10 [56, 57]. Higher scores indicate better HRQoL. Given Malaysia’s multiracial community, the SF-36 was administered in Malay, Mandarin, and English versions. As the reliability and validity of SF-36 instrument were previously examined in Malaysia [44], the reliability and validity of the Malay version of this generic HRQoL instrument were further confirmed, with a Cronbach alpha generally above 0.70 [44, 58, 59]. To date, there is no published study specifically evaluating the reliability and validity of the Mandarin version of the SF-36 within the Malaysian population. However, measurement equivalence between the English and Mandarin versions of the SF-36 has been established in Singapore, which is a multiethnic Asian country with demographic and linguistic profiles comparable to Malaysia [60]. Given this contextual similarity, and the common use of the SF-36 in Malaysian health research, the instrument was deemed appropriate for use in this study.

### Statistical analysis

Sociodemographic data of both informal caregivers and patients with AD were presented as mean (standard deviation (SD)) for continuous variables, and frequencies (percentage (%)) for categorical variables. Only complete responses without missing data were included in the statistical analysis. The scores in all eight subscales of SF-36 were calculated using scoring algorithms as well as the PCS and MCS. Histogram inspection and Shapiro Wilk test were used to determine data normality. As the distribution of the SF-36 domain scores were found normal from both graphical and statistical measures, independent t-test was performed to estimate the differences between caregivers’ scores and the general population in Malaysia.

Moreover, potential relationship between HRQoL scores (PCS and MCS) and sociodemographic factors of both patients and caregivers, and time spent on informal care was tested using univariate linear regression. To select variables to be inserted into multivariable analysis, the statistically significant value of the univariate linear regression was set at *p* < 0.25 [61]. Next, forward stepwise multiple linear regression analyses were performed with both PCS and MCS as different dependent variables. Categorical variables were dummy coded before entering into the model. Model assumptions were addressed with residual analyses and multicollinearity was checked with a cut-off point in variance inflation factor (VIF >10) and a tolerance value less than 0.20. All analyses were performed using Microsoft Excel and IBM Statistical Package for Social Sciences (SPSS) version 27 where the significance level for the multivariate regression models were set at *p* < 0.05 [62]. 

## Results

### Socio-demographic characteristics of AD patients and caregivers

During the study period, 161 informal caregivers were found eligible and recruited to the study. During the recruitment process, 25 of them refused to join the study, which led to 136 participants. After excluding 4 responses with missing data, 132 responses from informal caregivers of AD patients were included for analysis in the study. The mean age of patients was 80.75 ± 6.87 years, of which 72.7% of them were female as shown in Table 1. The majority of them (72%) were diagnosed with AD, where some of them (28%) were accompanies with other etiologies. Chinese ethnicity occupied a large proportion (81%) in this study population apart from Malay (6.82%), Indian (5.30%) and others (6.82%). Regarding their disease severity, most of the patients had moderate AD (40.20%) followed by mild (37.90%) and severe AD (22.00%). With a mean MMSE score of 16.38 ± 5.52, BPSD was only recorded in less than half (44.70%) of the patient study population. In terms of functional dependency, there was an increasing number of AD patients reported demand in BADL (66.67%), IADL (84.85%) and supervision (94.70%) along with time spent per month in BADL, IADL and supervision.

In terms of caregivers, they were mostly female (63.60%) with a mean age of 51.20 ± 13.50 years. Two thirds of the caregiver population were adult-child caregivers (67.50%) compared to spousal (13.60%). In addition, most of the caregivers were married (68.90%), living together with patients (70.45%) and working (68.20%). Only a few of them (16.70%) achieved an income level of M40 (RM5250 – RM11,819 per month) and 42% of them reported to have medical comorbidity. In addition, two thirds of informal caregivers provided care to patients more than 12 months (68.20%) where around one third (30.3%) of them were sole caregivers. As an alternative in providing care, some caregivers (15.9%) utilized SpA care in addition to standard informal care in community settings.

**Table 1:** Sociodemographic of caregivers and patients along with time spent on informal care in AD

Sociodemographic	Descriptives (*n* = 132)	
*Patient*		
Age in years (mean, SD)	80.75	6.87
Female gender (n, %)	96	72.70
Ethnicity (n, %)		
Malay	9	6.82
Chinese	107	81.06
Indian	7	5.30
Others	9	6.82
Diagnosis, (n, %)		
AD	95	72.00
Mixed	37	28.00
Disease severity (n, %)		
Mild	50	37.90
Moderate	53	40.20
Severe	29	22.00
Presence of BPSD (n, %)	59	44.70
MMSE Score (mean, SD)	16.38	5.52
Types of functional dependency, n (%)		
BADL	88	66.67
IADL	112	84.85
SV	125	94.70
Time spent on informal care, hr, (mean, SD)		
BADL (hr per month)	75.67	74.74
IADL (hr per month)	84.08	69.53
SV (hr per month)	156.44	110.20
*Informal Caregiver*		
Age in years, (mean, SD)	51.20	13.50
Female Gender (n, %)	84	63.60
Ethnicity (n, %)		
Malay	12	9.10
Chinese	105	79.50
Indian	7	5.30
Others	8	6.10
Relationship with patient (n, %)		
Spouse	18	13.60
Adult Child	89	67.50
Others	25	18.90
Cohabitation (n, %)	93	70.45
Marital Status (n, %)		
Married	91	68.90
Single/Divorce/Widow	41	31.10
No. of caregivers (n, %)		
Sole caregiver	40	30.30
More than one	92	69.70
Employment status (n, %)	90	68.20
Presence of medical comorbidities (n, %)	55	41.70
Duration of informal care, yr (n, %)		
≤ 12 months	42	31.80
> 12 months	90	68.20
Use of Special Accommodation (n, %)	21	15.90
Income level (n, %)		
B40 (≤ RM5,250)	110	83.30
M40 (RM5,251– RM11,819)	22	16.70
T20 (≥ RM11,820)	0	0.00

### SF-36 as measure of caregivers’ HRQoL

In this study, mean score of physical functioning (PF) was 82.92 ± 18.66; role limitations in physical (REP) 63.26 ± 43.62, role limitations in emotional (REE) 58.84 ± 44.33, vitality (VT) 50.80 ± 14.42, mental health (MH) 56.67 ± 14.39, social functioning (SF) 47.35 ± 11.35, bodily pain (BP) 27.10 ± 24.61, and general health (GH) 51.29 ± 10.96. With that, the PCS and MCS score of this study population were 51.18 ± 9.25 and 44.17 ± 11.19. Across disease severity, difference of SF-36 domain scores was neither statistically significant nor exhibiting trends as shown in Table 2.

**Table 2:** Mean SF-36 score of informal caregivers of alzheimer’s disease in different domains stratified by disease severity

Disease Severity (*n*)	Mild (*n* = 50)		Moderate (*n* = 53)		Severe (*n* = 29)		*p*-value	Total(*n* = 132)	
SF-36 items (score)	Mean	SD	Mean	SD	Mean	SD		Mean	SD
PF	83.80	19.50	81.42	18.12	84.14	18.62	0.75	82.92	18.66
REP	65.50	41.31	61.32	45.35	62.93	45.62	0.89	63.26	43.62
REE	62.67	43.46	49.69	45.11	68.97	42.66	0.13	58.84	44.33
VT	51.40	15.94	49.25	15.30	52.59	9.22	0.61	50.80	14.42
MH	53.84	15.10	57.74	14.83	59.59	11.68	0.95	56.67	14.39
SF	48.25	12.11	45.75	10.09	48.71	12.20	0.22	47.35	11.35
BP	20.89	20.41	30.82	25.00	31.03	28.84	0.07	27.10	24.61
GH	50.60	11.68	50.75	11.33	53.45	8.87	0.59	51.29	10.96
PCS	52.16	7.97	51.03	9.12	49.81	11.43	0.55	51.19	9.25
MCS	45.01	9.62	42.99	11.20	44.87	13.64	0.61	44.17	11.19

Remarks: *P* value for comparison of differences in means across AD severity groups using ANOVA

**Fig. 1:**
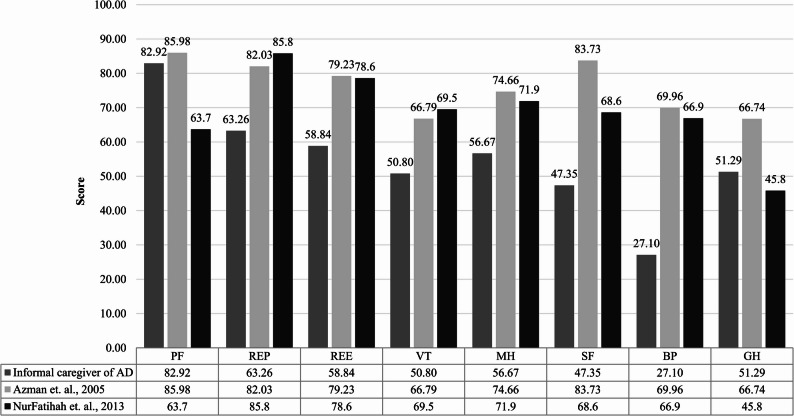
Comparison of caregivers’ health-related quality of life scores measured by MOS SF-36 with national population norm in Malaysia. Remarks: SF-36 36-item Short Form Health Survey; PF, Physical Functioning; REP, Role Limitations due to Physical Health; REE, Role Limitations due to Emotional Problems; VT, Vitality; MH, Mental Health; SF, Social Functioning; BP, Bodily Pain; GH, General Health

Figure 1 illustrated the results of eight domains in SF-36 compared with the population norm score obtained in previous study [63]. Compared to the population norm estimates, the mean scores of all eight SF-36 domain were significantly lower than that of general population norm scores. As there is lack of data regarding minimally clinical important differences (MCID) of SF-36 towards informal caregivers of AD, a cut-off value of 5 was utilized adapting from previous studies [64, 65]. In this study, most of the SF-36 domains has value reduction greater than 5 (ranged between − 15.45 and − 42.86) compared to population norm scores except PF (−3.06). On the other side, there are variations observed in the PCS, MCS and domain scores between this study population and dementia caregivers from Sarawak [66]. 

### Factors associated with caregivers’ HRQoL

The univariate analysis revealed that demand in BADL (crude B=−5.13, *p* < 0.01), demand in IADL (crude B=−4.54, *p* = 0.04), demand in SV (crude B=−4.18, *p* = 0.25), time spent in BADL monthly (crude B=−0.03, *p* < 0.01), time spent in IADL monthly (crude B=−0.02, *p* = 0.10), caregivers’ age (crude B=−0.201, *p* < 0.01), caregiver female gender (crude B=−4.29, *p* = 0.01), spousal relationship with patient (crude B=−3.95, *p* = 0.17), employment status (crude B = 2.49, *p* = 0.15), caregivers’ comorbidity (crude B=−4.47, *p* < 0.01), and duration of care more than 12 months (crude B=−2.02, *p* = 0.24) were associated with the PCS as shown in Table 3. With another dependent variable (MCS), the potential factors were shown in Table 4, including patient female gender (crude B = 5.75, *p* < 0.01), patient Indian ethnicity (crude B=−9.24, *p* = 0.10), demand in IADL (crude B=−3.37, *p* = 0.22), caregiver Indian ethnicity (crude B=−7.81, *p* = 0.18), spousal relationship with patient (crude B=−4.09, *p* = 0.24), and duration of care more than 12 months (crude B = 3.652, *p* = 0.08).

The forward stepwise multiple linear regression model assessed the association between PCS, MCS and the selected independent factors, as shown in Table 3 and Table 4. Due to multicollinearity between both patient and caregivers’ ethnicity, only caregivers’ ethnicity was selected if both factors showed significance in univariate analysis. From the multivariate models, caregiver age (standardised β=−0.30, *p* < 0.001), demand in BADL (standardised β=−0.25, *p* = 0.002) and caregivers’ female gender (standardised β=−0.19, *p* = 0.017) were found to negatively associated with PCS. Furthermore, only one independent variable namely patient female gender (standardised β = 0.23, *p* = 0.008) was found significant in the MCS multivariable analysis.

**Table 3:** Results of univariable and multivariable analysis with the physical component summary (PCS) of SF-36 as the dependent variable

		Univariable	95% CI			Multivariable^#^	95%CI		
Independent Variables	Category	Crude Beta	Lower	Upper	*p*-value	Crude Beta	Lower	Upper	*p*-value
*Constant*		-	-	-	-	67.16	60.86	73.45	< 0.001
*Patient-related factors*									
Age		−0.04	−0.27	0.20	0.757				
Gender	Male	R							
	Female	−1.59	−5.17	1.99	0.380				
Race	Malay	−1.24	−9.92	7.43	0.777				
	Chinese	1.23	−5.16	7.61	0.705				
	Indian	4.19	−5.09	13.46	0.373				
	Others	R							
Etiology	Mixed	R							
	AD	0.92	−2.63	4.48	0.609				
Severity	Mild	R							
	Moderate	−1.13	−4.75	2.48	0.536				
	Severe	−2.35	−6.63	1.93	0.280				
BPSD	No	R							
	Yes	1.51	−1.69	4.71	0.353				
MMSE Score		−0.06	−0.36	0.23	0.660				
*Disease-related factor*									
BADL	No	R				R			
	Yes	−5.13	−8.40	−1.85	**0.002**	−4.90	−7.98	−1.81	**0.002**
IADL	No	R							
	Yes	−4.54	−8.93	−0.16	**0.042**				
SV	No	R							
	Yes	−4.18	−11.27	2.92	**0.246**				
Monthly BADL time		−0.03	−0.05	−0.01	**0.005**				
Monthly IADL time		−0.02	−0.04	0.00	**0.098**				
Monthly SV time		0.00	−0.02	0.01	0.504				
*Caregiver-related factors*									
Age		−0.20	−0.31	−0.09	**0.001**	−0.20	−0.31	−0.09	**< 0.001**
Gender	Male	R				R			
	Female	−4.29	−7.53	−1.05	**0.010**	−3.69	−6.71	−0.67	**0.017**
Race	Malay	0.29	−8.13	8.71	0.946				
	Chinese	0.47	−6.30	7.24	0.891				
	Indian	3.65	−5.90	13.20	0.451				
	Others	R							
Relationship	Spouse	−3.95	−9.58	1.67	**0.167**				
	Adult Child	0.50	−3.62	4.61	0.812				
	Others	R							
Marital Status	Single/Divorce/Widowed	R							
	Married	1.04	−2.41	4.49	0.553				
Employment	No	R							
	Yes	2.49	−0.92	5.89	**0.151**				
Cohabitation	No	R							
	Yes	−0.14	−3.65	3.36	0.935				
Use of SpA	No	R							
	Yes	−0.34	−4.71	4.03	0.878				
Medical comorbidity	No	R							
	Yes	−4.47	−7.62	−1.32	**0.006**				
Number of CG	Sole	R							
	>One	−1.07	−4.55	2.40	0.541				
Duration of care	≤ 12 months	R							
	> 12 months	−2.02	−5.44	1.39	**0.243**				
Income level	B40 (≤ RM5250)	−1.04	−5.33	3.25	0.632				
	M40 (RM5,251 – RM11,819)	R							

Remarks: Independent variables with *p* < 0.25 were marked bold and selected into multivariable analysis. ^#^Model statistics: *R* = 0.442, Adj R2 = 0.177; F = 10.363; CI Confidence Interval; R denotes reference group; AD Alzheimer’s Disease; BPSD Behavioural and Psychological Symptoms in Dementia; MMSE Mini-Mental State Examination; BADL Basic Activity of Daily Living; IADL Instrumental Activity of Daily Living; SV Supervision; SpA Special Accommodation

**Table 4:** Results of univariable and multivariable analysis with the mental component summary (MCS) of SF-36 as the dependent variable

		Univariable	95% CI			Multivariable ^ф^	95% CI		
Independent Variables	Category	Crude B	Lower	Upper	*p*-value	Crude B	Lower	Upper	*p*-value
*Constant*		-	-	-	-	39.98	36.38	43.59	< 0.001
*Patient-related factors*									
Age		0.05	−0.23	0.33	0.740				
Gender	Male	R				R			
	Female	5.75	1.52	9.97	**0.008**	5.75	1.52	9.97	**0.008**
Race	Malay	−4.45	−14.90	5.99	0.401				
	Chinese	−4.36	−12.05	3.33	0.265				
	Indian	−9.24	−20.41	1.92	**0.104**				
	Others	R							
Etiology	Mixed	R							
	AD	−1.71	−6.00	2.59	0.432				
Severity	Mild	R							
	Moderate	−2.02	−6.40	2.36	0.362				
	Severe	−0.14	−5.33	5.05	0.957				
BPSD	No	R							
	Yes	−2.11	−5.98	1.76	0.284				
MMSE Score		0.11	−0.24	0.46	0.527				
*Disease-related factor*									
BADL	No	R							
	Yes	−0.77	−4.87	3.33	0.712				
IADL	No	R							
	Yes	−3.37	−8.73	1.99	**0.216**				
SV	No	R							
	Yes	1.52	−7.11	10.14	0.728				
Monthly BADL time		0.01	−0.01	0.04	0.386				
Monthly IADL time		−0.01	−0.04	0.01	0.315				
Monthly SV time		0.01	−0.01	0.03	0.352				
*Caregiver-related factors*									
Age		0.02	−0.13	0.16	0.833				
Gender	Male	R							
	Female	−0.32	−4.34	3.70	0.875				
Race	Malay	−1.42	−11.56	8.72	0.782				
	Chinese	−2.95	−11.09	5.20	0.476				
	Indian	−7.81	−19.30	3.69	**0.181**				
	Others	R							
Relationship	Spouse	−4.09	−10.94	2.77	**0.240**				
	Adult Child	−0.75	−5.77	4.27	0.768				
	Others	R							
Marital Status	Single/Divorce/Widowed	R							
	Married	−0.18	−4.35	4.00	0.934				
Employment	No	R							
	Yes	1.31	−2.84	5.45	0.533				
Cohabitation	No	R							
	Yes	−0.69	−4.93	3.55	0.747				
Use of SpA	No	R							
	Yes	0.17	−5.12	5.46	0.949				
Medical comorbidity	No	R							
	Yes	−1.71	−5.62	2.20	0.388				
Number of CG	Sole	R							
	>One	−0.31	−4.52	3.90	0.884				
Duration of care	≤ 12 months	R							
	> 12 months	3.65	−0.45	7.76	**0.081**				
Income level	B40 (≤ RM5250)	−0.67	−5.86	4.51	0.797				
	M40 (RM5,251 – RM11,819)	R							

Remarks: Independent variables with *p* < 0.25 were marked bold and selected into multivariable analysis. ^ф^ Model statistics: *R* = 0.23, Adj R2 = 0.046; F = 7.246; *p* = 0.008; CI Confidence Interval; R denotes reference group; AD Alzheimer’s Disease; BPSD Behavioural and Psychological Symptoms in Dementia; MMSE Mini-Mental State Examination; BADL Basic Activity of Daily Living; IADL Instrumental Activity of Daily Living; SV Supervision; SpA Special Accommodation

## Discussion

This present study utilized SF-36 health survey to examine the HRQoL of informal caregivers for patients with AD in both physical and mental aspects of well-being. Compared to caregivers from other regions, participants in this study exhibited higher scores in both domains than those reported in Japan, the United States, and several European countries [22, 33]. Such differences in perceived well-being could be explained by variations in cultural and societal values. Traditionally in Asian society, caregiving for older person is part of family responsibilities in their life instead of treating it as a burden [15, 17, 38, 67]. Besides, all subscales of SF-36 scores showed significant decrease among informal caregivers relative to the general population except the domain of PF. The pronounced disparity between AD caregivers and non-caregivers aligns with findings from previous studies, underscoring the critical need for caregiver support [16, 27, 33, 35, 38, 40, 41] However, the previous SF-36 scores observed in dementia caregivers from Sarawak, Malaysia indicated a greater decline in SF-36 scores than observed in the present study [66]. Given that the previous study was limited to a single region in Malaysia, its findings should be interpreted with caution due to potential limitations in national representativeness.

By providing BADL assistance to AD patients, a decline in PCS of caregivers was observed in this study which was consistent with previous literatures [28, 33, 68] It is not uncommon that reduced HRQoL scores were attained by informal caregivers of AD as great amount of time, energy and resources were devoted to provide BADL care to this vulnerable group [16, 20, 21, 30, 31]. In this study, over half of the respondents reported demand in BADL, such as bathing and eating, and IADL, such as running errands, managing finances and taking medicine, which were coherent with previous literature [13, 52]. As increasing time is spent providing care to AD patients, informal caregivers faced difficulty in managing work expectations and social relationships [14, 16, 20, 27]. In addition, great physical demand and strength are expected in family carers providing BADL care which further increases care burden [36, 40, 45]. With that, health deterioration could be anticipated with extended care demand and duration [14, 35, 36, 69]. 

As most of our caregivers in the study were over 50 years old, their physical endurance was not comparable with those in their 30–40 s [14]. A negative association was observed between caregivers’ age and HRQoL scores, particularly in the physical domain. As it was found consistent with a review, it was likely due to reduced engagement in regular physical activities as a result of caregiving responsibilities [48] [70]. As time is devoted to informal care, it eventually compromises their own free time in participating activities and causes social isolation [20]. Additionally, the high prevalence of comorbidities among older caregivers further compromises their health, increasing physical vulnerability [14, 24, 35]. With that, caregivers’ burden is anticipated to increased which also negatively influence their HRQoL [22, 71, 72]. Additionally, the high prevalence of comorbidities among older caregivers further compromises their health, increasing physical vulnerability [48]. Previous literatures had demonstrated specific favourable effects of various psychological interventions towards informal care of AD [73]. With better self-efficacy in managing demands from AD patients, informal caregivers could attain improved well-being [36]. Future studies could explore the impact of psychological resilience and coping mechanisms to understand their long-term role in caregiving experience, burden and well-being.

Furthermore, gender was found to significantly influence both the physical and mental dimensions of caregivers’ HRQoL, which was consistent with previous literature [48]. A negative association was observed between female caregivers and their physical HRQoL, consistent with prior research indicating lower quality of life among female dementia caregivers [15, 33, 74]. Good physical health is required to provide adequate care towards AD patients [45]. However, female caregivers, who often balance work and family responsibilities, are more vulnerable to adverse effects such as sleep disturbances, poor nutritional status, and neglect of self-care needs [75–77]. A cross-sectional study in China identified time-dependent burden as a significant challenge for female caregivers due to caregiving responsibilities [78]. This burden has been linked to a higher prevalence of depression among female caregivers compared to their male counterparts [79]. These findings underscore the need to consider gender differences in the design of social support programs, alongside cultural factors. Inequities in formal support services have been shown to contribute to reduced HRQoL among female caregivers [80]. Interestingly, in this study, providing care for female AD patients was positively associated with caregivers’ mental HRQoL, though this finding contrasts with previous research from other countries [30, 77]. Further studies are needed to explore the complex relationship between gender and perceived well-being in both physical and mental health domains.

The non-significant associations between caregiver HRQoL and factors such as relationship to the patient, employment status, caregiver comorbidity, and duration of care underscore the multifaceted nature of HRQoL determinants. While some prior research suggested that spousal caregivers may experience greater emotional and physical strain, its association with caregivers’ HRQoL was not observed significant in this study population [18, 35, 41, 72]. This was found consistent with previous reviews that urged more studies on this unclear association [48, 81]. On the other side, the close kinship of relationship was more pronounced as a predictor of caregivers’ HRQoL as observed in Thailand, which future studies are warranted [82]. Likewise, employment status was observed not significant in the multivariable model of caregivers’ HRQoL, which contrasts with previous findings [18, 27, 36].A cross-sectional study in Japan reported diminished HRQoL among employed young caregivers due to the challenges of managing work, family and caregiving responsibilities [27]. However, improved quality of life was observed in local Muslim caregivers from a recent study [36]. Despite the shared cultural value of filial piety in these Asian societies, the perception in care obligation may vary across populations [36, 83]. Future studies should explore these contextual variations to better understand the interplay between cultural expectations and caregiving experiences in shaping HRQoL.

Furthermore, the lack of association between caregiver comorbidity and HRQoL, which was contrasting with previous evidence that exhibited a negative association [18].This may suggest the role of resilience, self-care practices, or healthcare accessibility in mitigating the negative consequence of caregiving towards the physical and mental well-being [84, 85].As a mediator factor, caregivers’ self-efficacy was found to influence own HRQoL positively with the availability of social support [48, 82, 86]. In the univariable model, the positive association between long duration of care and caregivers’ mental HRQoL was observed despite of non-significance in further analysis. Nevertheless, this finding was found contradicting with previous results [15, 77]. In a local study among dementia caregivers, the mean duration of care was recorded 4.2 years, which was much greater than in this study population [15]. As this study analysed this factor in categorical terms, this methodological difference renders difficulty in cross-comparison with past findings. However, this may indirectly reflect a psychological adaptation process, where long-term caregivers develop effective coping strategies, or it may indicate the presence of external support, such as respite care, which helps maintain HRQoL [48, 83, 85, 87]. From LMIC, these findings highlight the importance of addressing protective factors, such as coping strategies, social support and respite care, in future research and interventions aimed at sustaining caregivers’ HRQoL rather than focusing solely on the burden imposed by caregiving-related factors [18].

### Strength

With the findings of this study, we gained more insights regarding the caregivers’ perceptions of HRQoL in providing informal care to patients with AD in physical and mental aspects which has rarely been explored in Malaysia. This is important for healthcare stakeholders in formulating healthcare policies for the older population as this study reflects the current scenario of caregivers providing informal care to patients with AD in Malaysia. By complementing previous estimates, this study provides updated insights into caregivers’ HRQoL, offering a clearer understanding of caregiving trends and the factors influencing their well-being. These findings enable policymakers to design targeted social and financial support schemes tailored to this vulnerable group, ensuring that caregivers receive the necessary assistance to sustain their roles effectively. Recognizing caregivers’ needs is essential for maintaining sustainable healthcare in AD. Strengthening community-based care for AD patients while safeguarding caregivers’ well-being requires coordinated policies that address financial relief, respite care, and psychosocial support, ultimately preventing caregiver burden and ensuring long-term care sustainability.

### Limitations

However, there are some limitations in this study. First, this research was cross-sectional in nature which precludes the casual relationships between variables. Future research employing a longitudinal study design is warranted to further explore these relationships over time. Second, the generalisability of the results may be limited across the nation as there was no data collected from southern Malaysia. In addition, the study population was mainly Chinese ethnicity despite Malay as the dominant ethnicity in Malaysia. According to a 2021 study, Malay are less likely to have medical check-ups than Chinese and Indian among the aging population [88]. This could partly explain the ethnicity differences in this study. Next, the degree of social support was not investigated in this study via validated tools which render difficulty in understanding its potential impact towards caregivers’ well-being. Although variables such as number of caregivers involved and use of special accommodation care were sought in this study, it could not directly reflect the extent of support perceived by caregivers in this study. Future research could consider to measure the availability and extent of social support received in order to assess potential associations with AD caregivers’ well-being.

## Conclusion

Compared to general population, a marked decline in both physical and mental aspects of HRQoL was recorded among informal caregivers of AD in Malaysia. Demands in BADL and increasing caregivers age caused physical strain on informal caregivers, which reflected as reduced PCS. Female caregivers reported a reduced score in physical HRQoL while female patient was associated with greater MCS in this study population. These findings underscore the necessity for support programs that address physical care demands and provide psychosocial interventions, particularly for older and female caregivers. Longitudinal research is necessary in Asian communities to gain more understanding on the impact of caregiver’s socio-demographics, protective factors and disease-related factors toward caregivers’ well-being.

## Data Availability

The datasets used and/or analysed during the current study are available from the corresponding author on reasonable request.
